# Editorial: Thyroid hormone and metabolites: central versus peripheral effects, volume II

**DOI:** 10.3389/fendo.2023.1241233

**Published:** 2023-07-18

**Authors:** Pieter de Lange, Federica Cioffi, Rosalba Senese, Grazia Chiellini

**Affiliations:** ^1^ Dipartimento di Scienze e Tecnologie Ambientali, Biologiche e Farmaceutiche, Università della Campania “Luigi Vanvitelli”, Caserta, Italy; ^2^ Dipartimento di Scienze e Tecnologie, Università del Sannio, Benevento, Italy; ^3^ Dipartimento di Patologia Chirurgica, Medica, Molecolare e dell’Area Critica, Università di Pisa, Pisa, Italy

**Keywords:** thyroid hormones, thyroid-stimulating hormone, thyroid dysfunctions, cardiovascular disease, metabolic disorders, COVID-19 disease

Thyroid hormones (THs) produce a wide variety of physiological effects in virtually all tissues and metabolic pathways. The main hormones produced by the thyroid gland are thyroxine or tetraiodothyronine (T4) and triiodothyronine (T3). In cells, TH actions are mediated mainly by nuclear TH receptors (THR), which modify gene expression. T3 is the preferred ligand of THR, whereas T4, the serum concentration of which is 100-fold higher than that of T3, undergoes extra-thyroidal conversion to T3. This conversion is catalyzed by 5′-deiodinases (D1 and D2), which are TH-activating enzymes. The regulation of deiodinases permits cell-targeted fine regulation of TH homeostasis, affecting metabolic pathways in health and disease states.

Maintenance of the circulating levels of THs is controlled by a self-regulatory circuit called the hypothalamic–pituitary–thyroid axis. Thyrotropin-releasing hormone (TRH) from the hypothalamus, thyroid-stimulating hormone (TSH) from the anterior pituitary gland, and T4 work in synchronous harmony to maintain a proper feedback mechanism and homeostasis. Deficiency of THs, caused by an underactive thyroid gland, namely, hypothyroidism, typically manifests as bradycardia, cold intolerance, constipation, fatigue, and weight gain. In contrast, excess of THs, caused by increased thyroid gland function, namely, hyperthyroidism, manifests as weight loss, heat intolerance, diarrhea, fine tremor, and muscle weakness.

The roles of THs are crucial to nervous system development, normal growth, energy expenditure, and thermogenesis.

THs have a complex relationship with the cardiovascular system through multiple mechanisms, and patients with severe hypothyroidism or hyperthyroidism are known to have an accelerated onset of cardiovascular disease (CVD).

Timely recognition and effective treatment of cardiac symptoms in patients with thyroid dysfunction are mandatory to prevent heart failure (HF), which represents a major cause of morbidity and mortality in Europe and the United States.

In the present Research Topic, Lang et al. investigated the probable relationship between free triiodothyronine (FT3), free tetra-iodothyronine (FT4), or the FT3/FT4 ratio and all- cause cardiovascular mortality and CVD risk in the general population of the United States without previous thyroid illness using a representative national sample, namely, the NHANES 2007-12 database. By integrating and analyzing the data from the large database, they proposed that among adults in the United States, the FT3/FT4 ratio is associated with CVD and is an independent predictor of cardiovascular death and all-cause death. Therefore, they recommended examining the FT3/FT4 ratio in addition to the FT3 and FT4 concentrations in the general population.

The results of a systematic review and meta-analysis, reported by Zhang H. et al., highlighted that N-terminal pro-B-type natriuretic peptide (NT-pro-BNP), a cardiac neurohormone generated by ventricles in response to volume expansion or pressure overload, could represent a useful biomarker for diagnosing, evaluating, and predicting outcomes in heart failure (HF). Taking into account that the prognosis of HF may be improved with appropriate treatment of thyroid disfunction (TD), the authors suggested to perform randomized controlled trials to examine the prognosis and potential improvement in HF with appropriate treatment of TD.

As reported by Zhang M. et al., a large variety of studies in mouse models of hyperthyroidism have been carried out, and potential novel treatments for Graves’ disease (GD), the most common type of hyperthyroidism, have emerged, including a monoclonal antibody against intracellular adhesion molecule-1 (ICAM-1 mAb) and siRNA targeting the thyroid-stimulating hormone receptor (TSHR), as well as natural products such as fucoxanthin and icariin. Nevertheless, further studies using multiple models of the disease can provide additional therapeutic options.

As elegantly reviewed by Vieira et al., in addition to the well-established role of the TSHR in TH metabolism, the discovery of TSHR expression in various organs has led to a view of the TSH as a pleiotropic agent, which can act on several extrathyroidal diseases. The availability of new molecules with the function of TSHR agonists, antagonists, or inverse agonists can contribute to improving our understanding on the extrathyroidal roles of the TSHR.

Impaired sensitivity to THs has been demonstrated to be positively associated with the prevalence of metabolic disorders, including metabolic dysfunction-associated fatty liver disease (MAFLD). Currently, the exact relationship between thyroid function parameters (FT3, FT4, and TSH) and MAFLD remains controversial. Hu et al. reported the results of a retrospective cross-sectional study involving 177,540 individuals with thyroid function tests and MAFLD diagnosis from 2010-2018. Their findings suggest non-linear relationships between thyroid function parameters and MAFLD, emphasizing the need to extend the investigation to larger prospective studies with long-term follow-up.

In addition, it is becoming increasingly clear that thyroid function is associated with uric acid (UA) metabolism. Hyperuricemia (HUA) is not only a precursor of gout and kidney failure, but it may also contribute to the development of metabolic syndrome and CVD. Both epidemiologic and genetic research support the essential element of HUA in CVD progression. In their study, Lu et al. found that both impaired central and peripheral sensitivity to THs were associated with elevated UA levels in a Chinese euthyroid population. In addition, they observed that the associations of TH sensitivity indices with elevated UA levels were more apparent in female people than in male people, suggesting the potential impact of sex-related TH sensitivity on UA metabolism.

Numerous recent studies have focused on the role of pre-existing morbidities for risk of infection with SARS-CoV-2 and the severity of COVID-19 disease. Although TD appears to play no important role as a risk factor for contracting COVID-19 disease, alterations in TH levels were observed in hospitalized patients. By conducting a retrospective study in a large group of COVID-19 patients, Deng et al. found that the severe and non-survival patients with COVID-19 had a lower serum FT3. In addition, their investigation showed that FT3 was positively associated with Lys and ALB and negatively related to C-reactive protein (CRP), the erythrocyte sedimentation rate (ERS), and D-dimer, ultimately suggesting a significant predictive value of FT3 on COVID-19 prognosis and a significant relationship with the clinical parameters of inflammation, coagulopathy, and fibrinolysis.

Collectively, all the information provided by the contributions to this Research Topic will significantly enrich our understanding of the role of thyroid function in maintaining human health.

## Author contributions

PL, FC, and RS: contributions to the conception of the work; GC: contribution to the drafting of the work and of the final approval of the version to be published.

